# Association between SOFA score and risk of acute kidney injury in patients with diabetic ketoacidosis: an analysis of the MIMIC-IV database

**DOI:** 10.3389/fendo.2024.1462330

**Published:** 2024-12-23

**Authors:** Yiming Hua, Ning Ding, Huaizhi Jing, Yifei Xie, Hao Wu, Yue Wu, Beidi Lan

**Affiliations:** ^1^ Department of Cardiovascular Medicine, The First Affiliated Hospital of Xi’an Jiaotong University, Xi’an, Shaanxi, China; ^2^ Key Laboratory of Molecular Cardiology, Key Laboratory of Environment and Genes Related to Diseases, Ministry of Education, Xi’an Jiaotong University, Xi’an, Shaanxi, China; ^3^ Department of Cardiovascular Surgery, First Affiliated Hospital of Xi'an Jiaotong University, Xi'an, Shaanxi, China

**Keywords:** SOFA, diabetic ketoacidosis, acute kidney injury, MIMIC-IV database, ICU - intensive care unit

## Abstract

**Introduction:**

The Sequential Organ Failure Assessment (SOFA) score is a widely utilized clinical tool for evaluating the severity of organ failure in critically ill patients and assessing their condition and prognosis in the intensive care unit (ICU). Research has demonstrated that higher SOFA scores are associated with poorer outcomes in these patients. However, the predictive value of the SOFA score for acute kidney injury (AKI), a common complication of diabetic ketoacidosis (DKA), remains uncertain. Therefore, this study aims to investigate the relationship between SOFA scores and the incidence of AKI in patients with DKA.

**Methods:**

The study population was divided into two groups based on the median SOFA score (Q1: SOFA ≤3; Q2: SOFA >3). The primary endpoint was the incidence of AKI in patients with DKA. Secondary endpoints included renal replacement therapy (RRT) utilization and in-hospital mortality. Kaplan–Meier survival analysis, Cox proportional hazards models, and logistic regression models were employed to assess the association between SOFA and therisk of AKI in patients with DKA.

**Results:**

Overall, 626 patients with DKA were included in this study, of which 335 (53%) were male. Kaplan–Meier survival analysis included that patients with higher SOFA scores experienced significantly increased cumulative incidences of AKI, higher rates of RRT utilization, and elevated in-hospital mortality. Furthermore, after adjusting for confounding factors, logistic regression and Cox proportional hazards analyses confirmed that SOFA scores remained significantly associated with the incidence of AKI in patients with DKA.

**Conclusions:**

Our study indicates that a high SOFA score is an independent risk predictor for the occurrence of AKI, the utilization of RRT, and in-hospital mortality in patients with DKA. The sofa score can be utilized as a biomarker to assess the risk of AKI in this patient population.

## 1 Introduction

Diabetic ketoacidosis (DKA) is a common medical emergency in patients with diabetes, predominantly affecting those with uncontrolled type 1 diabetes mellitus (T1DM), but it can also occur in poorly managed type 2 diabetes mellitus (T2DM) due to certain medications. Various factors, such as infections, often act as triggers for DKA (1). DKA is characterized by severe hyperglycemia, metabolic acidosis, dehydration, and electrolyte imbalances (2), which can lead to serious complications, including cerebral edema, hypokalemia, and acute kidney injury (AKI) (3–5). AKI, a frequent complication, occurs in 40–50% of severe DKA cases, affecting both children (3) and adults, especially those admitted to intensive care units (6). The primary cause of DKA-related AKI (DKA-AKI) is inadequate renal perfusion resulting from hypovolemia. Therefore, identifying risk factors for DKA-AKI and understanding the characteristics of high-risk groups is crucial for early intervention and the prevention of AKI in these patients. The Sequential Organ Failure Assessment (SOFA) score is a widely recognized tool for assessing the severity of organ dysfunction across multiple systems, including the respiratory, cardiovascular, liver, coagulation, renal, and nervous systems (7). It is commonly used to predict outcomes in critically ill patients with conditions such as sepsis, trauma, and heart failure (8–10). In patients with DKA, systemic hypoperfusion and inflammatory responses can lead to multi-organ dysfunction, which aligns with the criteria evaluated by the SOFA score. However, the relationship between the SOFA score and the development of AKI, a severe complication of DKA, remains unclear. This study aims to explore the potential of the SOFA score as a predictive marker for the incidence of AKI in patients with DKA, with the goal of informing early intervention strategies to prevent this complication.

In this study, we employed machine learning techniques to identify the most significant predictors, including the SOFA score, and validated its role in predicting DKA-AKI through multivariate logistic regression models, Cox proportional hazards models, and subgroup analyses. Our findings demonstrate that a higher SOFA score is significantly associated with an increased risk of AKI in DKA patients. The assessment of the SOFA score at the time of admission is therefore critical, as it serves as an early warning signal for clinicians to anticipate the development of AKI, potentially improving clinical outcomes through timely preventive measures.

## 2 Materials and methods

### 2.1 Data source

This study utilizes the freely accessible Medical Information Mart for Intensive Care-IV (MIMIC-IV) database, which contains comprehensive information on all patients treated at the Beth Israel Deaconess Medical Center in Boston, Massachusetts from 2008 to 2019. The database includes vital signs, laboratory tests, medication treatments, risk assessment indicators, and other relevant information. Additionally, the first author, Yiming Hua, has obtained official certification from the database and has the necessary qualifications to access the data (ID: 52681986). The database de-identifies personal information by replacing patient identities with random codes. Therefore, the requirement for patient informed consent or ethical approval was waived.

**Figure 1:**
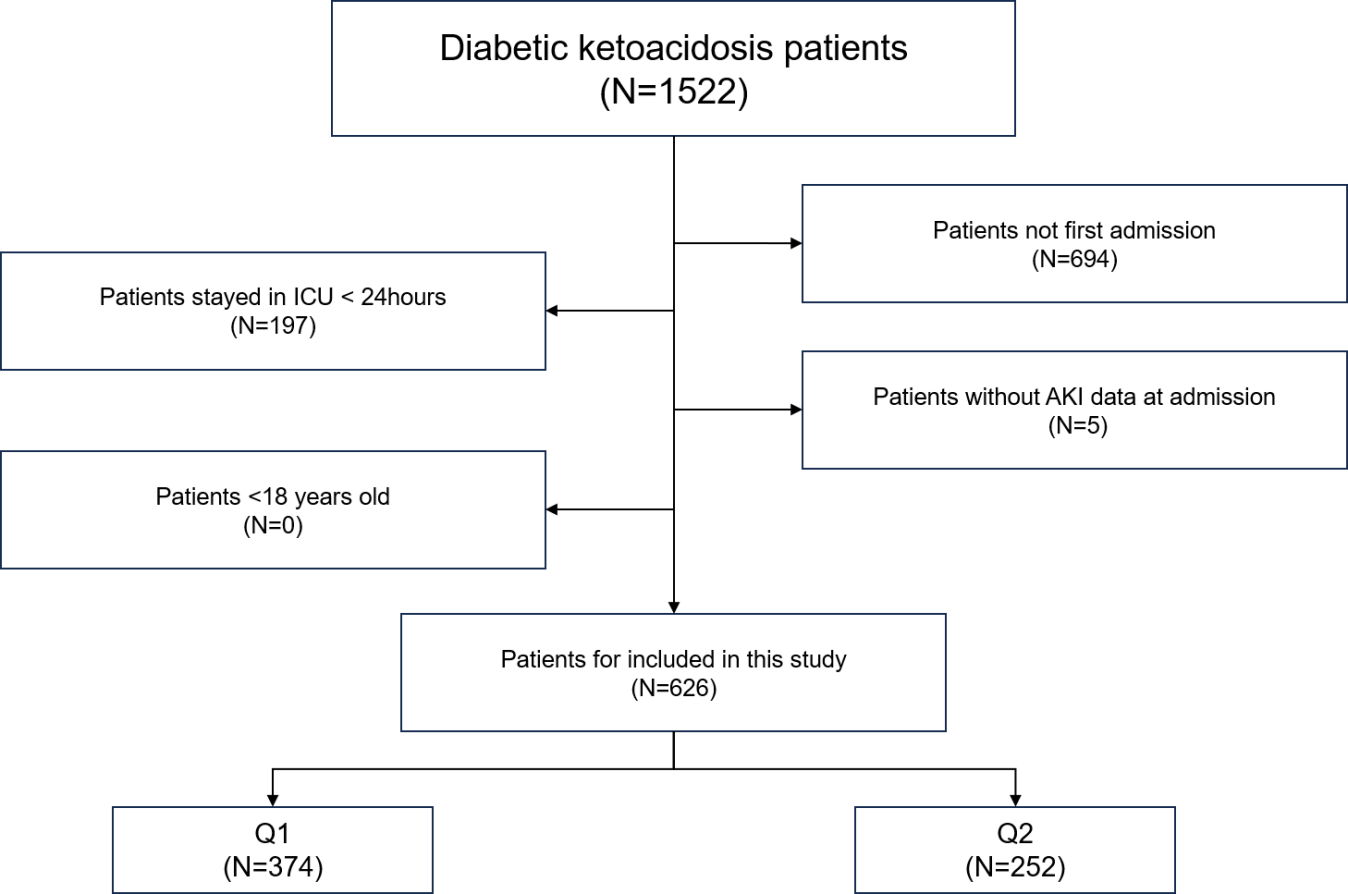
Overall flowchart of this study. MIMIC-IV, Medical Information Mart for Intensive Care IV.

Patients with DKA were identified in the MIMIC-IV database using the International Classification of Diseases (ICD-9/10) codes. The exclusion criteria were as follows: (1) patients with non-first hospital admission; (2) lack of assessment data for AKI; (3) patients with ICU stays less than 24 hours; (4) patients aged less than18 years (**Figure 1**).

### 2.2 Data extraction

We utilized the Structured Query Language to extract data from Navicat Premium software (version 12). The data, obtained from the MIMIC-IV database within 24 h of admission, included age, gender, body mass index (BMI), diabetes mellitus (DM) type, heart rate, systolic blood pressure, diastolic blood pressure, respiratory rate (RR), lymphocytes, neutrophils, platelets, white blood cells, red blood cells, hematocrit, hemoglobin, blood urea nitrogen (BUN), serum creatinine (SCR), glucose, anion gap, bicarbonate, calcium, chloride, sodium, potassium, lactate, albumin, PH, hypertension, coronary artery disease (CAD), chronic heart failure (CHF), chronic kidney disease (CKD), liver disease, chronic obstructive pulmonary disease (COPD), malignancy, and SOFA score. The follow-up period for this study commenced at the patient’s initial hospital admission and extended until the detection of AKI. Additionally, for the secondary endpoint, the follow-up period was extended from initial hospital admission to the first use of renal replacement therapy RRT.

In our study, variables with missing values exceeding 65% were excluded from the analysis, in accordance standards established in previous literature (11, 12). To handle the remaining missing data, we employed the “missForest” package in R to impute values prior to model fitting.

#### 2.2.1 Primary outcome and secondary outcomes

The primary endpoint of this study was the incidence rate of acute kidney injury (AKI). The diagnostic criteria for AKI were based on the Kidney Disease: Improving Global Outcomes (KDIGO) guidelines, which define AKI as either an increase in serum creatinine (SCR) to ≥1.5 times the baseline within 7 days, an increase of ≥0.3 mg/dL in SCR within 24 hours, or oliguria (13). The baseline SCR used in this study was the measurement obtained within the first 24 hours of admission. RRT served as an indirect indicator of AKI severity and in-hospital mortality and was considered a secondary endpoint in this study.

#### 2.2.2 Feature selection

Before investigating the impact of the SOFA score on the occurrence of acute kidney injury (AKI) in patients with diabetic ketoacidosis (DKA), we employed the Boruta algorithm, a machine-learning technique used for feature selection and assessing the importance of variables in predictive models (14). This algorithm generates shadow features based on the dataset and identifies variables that significantly influence the outcome. Additionally, we fitted a random forest model for variable selection and utilized the Shapley Additive Explanations (SHAP) package to visualize the importance of these variables (15). And we used the R packages (PerformanceAnalytics and Car) to assess multicollinearity among variables and calculated the variance inflation factor (VIF). A VIF threshold of less than 10 was applied to prevent overfitting in the model. The selected variables were subsequently included in our analyses using Cox proportional hazards and logistic regression models, which were essential for interpreting our results.

### 2.3 Statistical analysis

Continuous variables were presented as means and standard deviations, and between-group comparisons conducted using either the Mann–Whitney U test or Student’s t-test. Categorical variables were expressed as frequencies and percentages, and between-group comparisons were performed using Fisher’s exact test or Pearson’s chi-square test.

**Figure 2:**
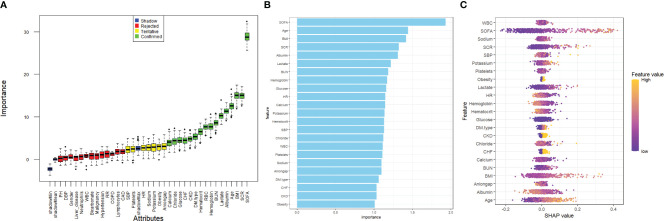
Application of machine learning in feature selection. **(A)** Feature selection for the relationship between various SOFA score and AKI was analyzed using the Boruta algorithm. **(B)** Variable Importance for Random Forest Model. **(C)** Shapley Additive Explanations (SHAP) for the random forest model. A distribution of the impact of each feature on the model output. Each dot represents a patient in a row. The colors of the dots represent the feature values: yellow represents larger values and purple represents lower values.

Kaplan–Meier survival analysis was employed to assess the correlation between the SOFA score and the incidence of acute kidney injury (AKI) in each group. Additionally, a Cox proportional hazards model analysis was performed to calculate the odds ratios (ORs) and their corresponding 95% confidence intervals (CIs) for the impact of the LAR index on AKI incidence across different groups. This analysis adjusted for multiple variables that may have influenced the outcomes. In Model 1, no variable adjustments were made. Model 2 included adjustments for gender, age, and body mass index (BMI). In Model 3, based on the results from feature importance selection using the Boruta algorithm and random forest model (as illustrated in **Figures 2A–C**), the following variables were included: gender, age, BMI, serum creatinine (SCR), lactate, albumin, blood urea nitrogen (BUN), red blood cell count (RBC), type of diabetes mellitus (DM), chronic kidney disease (CKD), chronic heart failure (CHF), glucose, chloride, and calcium. Each model incorporated the SOFA score in both continuous and categorical forms. The Q1 group served as the baseline group in all models. Furthermore, restricted cubic splines were applied to examine the relationship between SOFA scores and outcome events, including AKI, renal replacement therapy (RRT), and in-hospital mortality, based on the fully adjusted Model 3.

Subgroup analyses were conducted to explore the impact of the SOFA score on outcome indicators within various subgroups defined by sex (female vs. male), age (<65 vs. ≥65 years), DM type (T1DM, T2DM, and other), BMI (<30 vs. ≥30 kg/m²), and the presence of conditions, such as CHF, CKD, liver disease, and CAD.

All data analyses were performed using R version 4.2.3. A two-sided p-value <0.05 was considered statistically significant.

## 3 Results

### 3.1 Baseline characteristics

**Table 1** presents the baseline characteristics of patients with diabetic ketoacidosis (DKA), categorized by their SOFA scores (Q1: SOFA ≤3; Q2: SOFA >3). The mean SOFA scores for the two groups were 1.76 ± 0.94 and 7.60 ± 3.59, respectively. The incidence rates of AKI in these groups were 14.2% and 62.3%, respectively. In terms of demographic data, the Q2 group was older, had a higher BMI, a higher proportion of males, and a greater prevalence of T2DM, alongside an elevated respiratory rate. In the laboratory tests, the Q2 group had lower lymphocyte percentage, platelets, RBC, hematocrit percentage, hemoglobin, and albumin levels than the Q1 group. However, they have higher white blood cells, BUN, SCR, glucose, sodium, lactate, and PH levels. Additionally, the prevalence of comorbidities such as CHF, COPD, CKD, liver disease, and CAD was significantly higher in the Q2 group (all, p <0.05).

**Table 1:** Baseline characteristics according to SOFA score.

	Q1	Q2	
	N=(374)	N=(252)	Pvalue
Demographic			
Age	45.93 ± 17.95	60.25 ± 15.60	<0.001
Gender			
Male	177 ( 47.3)	158 ( 62.7)	<0.001
Female	197 ( 52.7)	94 ( 37.3)	
BMI	26.48 ± 7.10	29.39 ± 7.27	0.002
DM type			<0.001
T1DM	233 ( 62.3)	101 ( 40.1)	
T2DM	114 ( 30.5)	110 ( 43.7)	
Other	27 (7.2)	41 ( 16.3)	
Vital Signs			
HR (beats/min)	92.31 ± 13.94	91.35 ± 17.16	0.459
SBP (mmHg)	123.19 ± 16.02	121.51 ± 19.66	0.259
DBP (mmHg)	92.60 ± 17.56	91.88 ± 19.60	0.64
RR,breaths/min	28.22 ± 6.43	29.79 ± 7.15	0.005
Laboratory tests			
Lymphocytes,%	12.87 ± 9.26	10.88 ± 10.91	0.031
Neutrophils,%	80.05 ± 11.38	80.97 ± 13.45	0.42
Platelets (K/uL)	270.55 ± 97.61	220.20 ± 104.74	<0.001
WBC (K/uL)	12.45 ± 5.75	14.19 ± 6.57	0.001
RBC (K/uL)	4.10 ± 0.70	3.73 ± 0.80	<0.001
Hematocrit,%	36.48 ± 5.83	33.52 ± 6.30	<0.001
Hemoglobin (g/dL)	12.14 ± 2.06	11.00 ± 2.13	<0.001
BUN (mg/dL)	24.09 ± 14.06	50.22 ± 28.63	<0.001
SCR (mg/dL)	1.25 ± 0.51	3.06 ± 2.48	<0.001
Glucose (mg/dL)	204.54 ± 40.19	229.86 ± 62.85	<0.001
Aniongap (mEq/L)	27.06 ± 7.75	26.60 ± 7.97	0.466
Bicarbonate (mEq/L)	20.98 ± 4.34	21.48 ± 4.57	0.173
Calcium (mg/dL)	8.93 ± 0.88	8.95 ± 1.13	0.811
Chloride (mEq/L)	109.91 ± 6.50	109.58 ± 8.72	0.612
Sodium (mEq/L)	140.22 ± 4.62	142.48 ± 7.93	<0.001
Potassium (mEq/L)	5.16 ± 1.17	5.32 ± 1.17	0.096
Albumin (g/dL)	3.91 ± 0.75	3.39 ± 0.69	<0.001
Lactate (mmol/L)	2.25 ± 1.57	3.92 ± 3.61	<0.001
PH	7.34 ± 0.11	7.37 ± 0.11	0.002
Comorbidities,n(%)			
CHF	32 (8.6)	64 ( 25.4)	<0.001
COPD	39 ( 10.4)	53 ( 21.0)	<0.001
CKD	47 ( 12.6)	104 ( 41.3)	<0.001
Malignancy	14 (3.7)	18 (7.1)	0.087
Liver disease	22 (5.9)	34 ( 13.5)	0.002
Hypertension	132 ( 35.3)	89 ( 35.3)	1
Obesity	34 (9.1)	36 ( 14.3)	0.058
CAD	44 ( 11.8)	65 ( 25.8)	<0.001
**AKI**	53 ( 14.2)	157 ( 62.3)	<0.001
**SOFA**	1.76 ± 0.94	7.60 ± 3.59	<0.001

BMI, Body Mass Index; HR, heart rate; SBP, systolic blood pressure; DBP, diastolic blood pressure; WBC, white blood cells; RBC, red blood cells; BUN, blood urea nitrogen; SCR, serum creatinine; CAD, coronary artery disease; CHF, chronic heart failure; CKD, chronic kidney disease; COPD, Chronic obstructive pulmonary disease; diabetes; SOFA, sequential organ failure assessment; AKI, Acute kidney injury.

### 3.2 Outcomes

**Figure 3:**
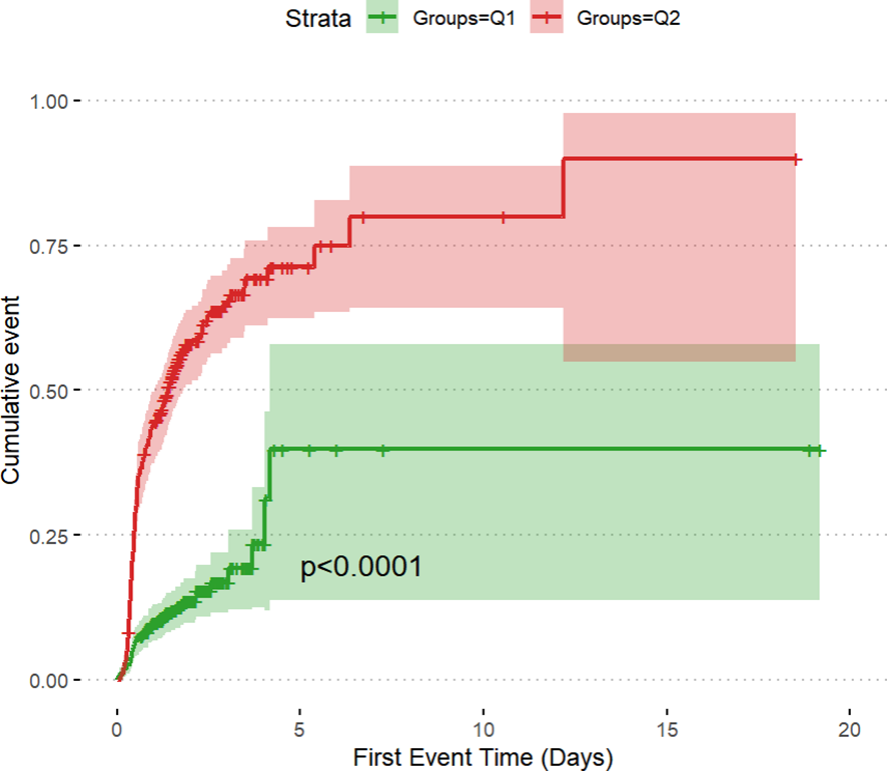
The cumulative event incidence curves for incidence of AKI.

**Figure 4:**
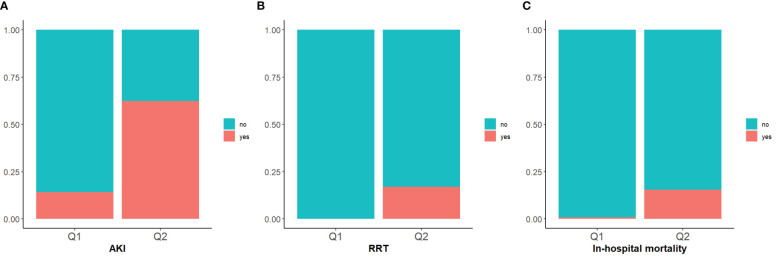
Stacked percent bar chart of AKI, RRT, in-hospital mortality. **(A–C)**: The incidence of AKI, RRT use, and in-hospital mortality are shown separately.

The incidence rates of AKI among the two groups of patients with DKA were 14.2% in Q1 and 62.3% in Q2, underscoring the increased risk of AKI in the Q2 group with higher SOFA scores (**Table 2**). The cumulative risk curve also indicated that during the follow-up period, the incidence of AKI in the Q2 group increased (p <0.0001) (**Figures 3**, **4**). Additionally, the in-hospital mortality rate in the Q2 group was 15.5%, in contrast to 0.8% in the Q1 group (**Figure 4**). Notably, all patients who required renal replacement therapy (RRT) were from the Q2 group, totaling 43 individuals, which constituted 17.1% of that group. Most of these patients commenced RRT within the first three days of hospital admission (**Table 2**; **Supplementary Figure S1**). All p-values were below 0.05.

**Table 2:** Outcome events.

	Q1	Q2	
	N=(374)	N=(252)	Pvalue
AKI	53 ( 14.2)	157 ( 62.3)	<0.001
RRT	0 (0.0)	43 ( 17.1)	<0.001
In-hospital mortality	3 (0.8)	39 ( 15.5)	<0.001
Los_icu	2.03 ± 1.01	5.11 ± 5.44	<0.001
Los_hospital	6.14 ± 6.12	11.82 ± 10.91	<0.001

RRT, renal replacement therapy; Los_hospital, length of stay in hospital; Los_icu, length of stay in ICU.

Multivariate logistic regression analysis demonstrated that SOFA scores were independently associated with AKI incidence, RRT usage, and in-hospital mortality. When SOFA is treated as a categorical variable, in the fully adjusted Model 3 indicated that the risks of AKI incidence and in-hospital mortality for the Q2 group are as follows: hazard ratio [HR], 3.45 (95% CI: 2.067–5.741); and HR, 7.453 (95% CI: 1.938–28.666), respectively. When SOFA is treated as a continuous variable, the risks of AKI incidence, RRT usage, and in-hospital mortality are as follows: HR, 1.345 (95% CI: 1.228–1.473); HR, 1.449 (95% CI: 1.254–1.674); and HR, 1.436 (95% CI: 1.263–1.633), respectively (**Table 3**). Furthermore, the Cox proportional hazards model assessed the predictive risk of AKI incidence among patients with DKA based on SOFA scoring, yielding HRs in the fully adjusted Model 3 of 2.320 (95% CI: 1.587–3.393) for categorical SOFA scores and 1.153 (95% CI: 1.105–1.203) when SOFA scores were treated as categorical and continuous variables, respectively (**Supplementary Table S1**).

**Table 3:** Logistic regression model of SOFA and AKI, RRT, and in-hospital mortality.

Categories	Model 1		Model 2		Model 3	
	HR (95%CI)	Pvalue	HR (95%CI)	Pvalue	HR (95%CI)	Pvalue
AKI incidence						
SOFA as continuous	1.543 [95%CI 1.430-1.664]	<0.001	1.461 [95%CI 1.350-1.583]	<0.001	1.345 [95%CI 1.228-1.473]	0.009
Quartile^a^						
Q1	Ref.		Ref.		Ref.	
Q2	10.009 [95%CI 6.800-14.731]	<0.001	7.169 [95%CI 4.696-10.946]	<0.001	3.445 [95%CI 2.067-5.741]	<0.001
RRT usage						
SOFA as continuous	1.370 [95%CI 1.271-1.478]	<0.001	1.412 [95%CI 1.297-1.538]	<0.001	1.449 [95%CI 1.254-1.674]	<0.001
In-hospital mortality						
SOFA as continuous	1.435 [95%CI 1.321-1.559]	<0.001	1.431 [95%CI 1.307-1.566]	<0.001	1.436 [95%CI 1.263-1.633]	<0.001
Quartile^a^						
Q1	Ref.		Ref.		Ref.	
Q2	22.643 [95%CI 6.914-74.155]	<0.001	17.330 [95%CI 5.100-58.888]	<0.001	7.453 [95%CI 1.938-28.666]	0.003

Model 1 was unadjusted.

Model 2 was adjusted for gender, age, and BMI.

Model 3 was adjusted for gender, age, BMI, SCR, lactate, albumin, BUN, RBC, DM type, CKD, CHF, glucose, chloride, and calcium.

The lowercase letter 'a' following 'Quartile' indicates that, according to the research method described earlier, the two groups were divided based on the 50% quartile.

**Figure 5:**
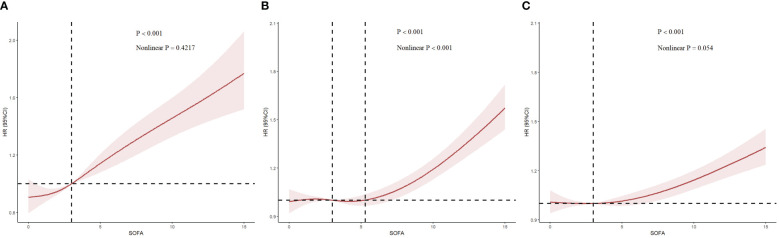
Restricted cubic spline (RCS) showing the relationship between SOFA and outcome indicators. **(A–C)**: RCS showed the correlation between SOFA and AKI, in-hospital mortality and RRT on the fully adjusted Logistic regression model, respectively. Nonlinear P indicated whether SOFA had a linear correlation with the outcome, and P>0.05 indicated a linear correlation.

**Figure 5** illustrates the restricted cubic splines regression model based on the fully adjusted logistic regression of Model 3, highlighting the dose-response relationship between SOFA scores and risks of AKI incidence, in-hospital mortality, and RRT usage (nonlinear p = 0.4217, nonlinear p <0.001, and nonlinear p = 0.054, all p <0.001). The restricted cubic spline regression models for AKI and in-hospital mortality demonstrate a clear positive correlation with SOFA scores, while the model for RRT suggested a hyperbolic relationship, indicating that the risk of RRT usage increases with higher SOFA scores when the score exceeds 3. Additionally, we employed the restricted cubic spline regression model based on the fully adjusted Cox proportional hazards model of Model 3 to evaluate the relationship between SOFA and AKI, which corroborated the previously mentioned results (**Supplementary Figure S2**).

**Figure 6:**
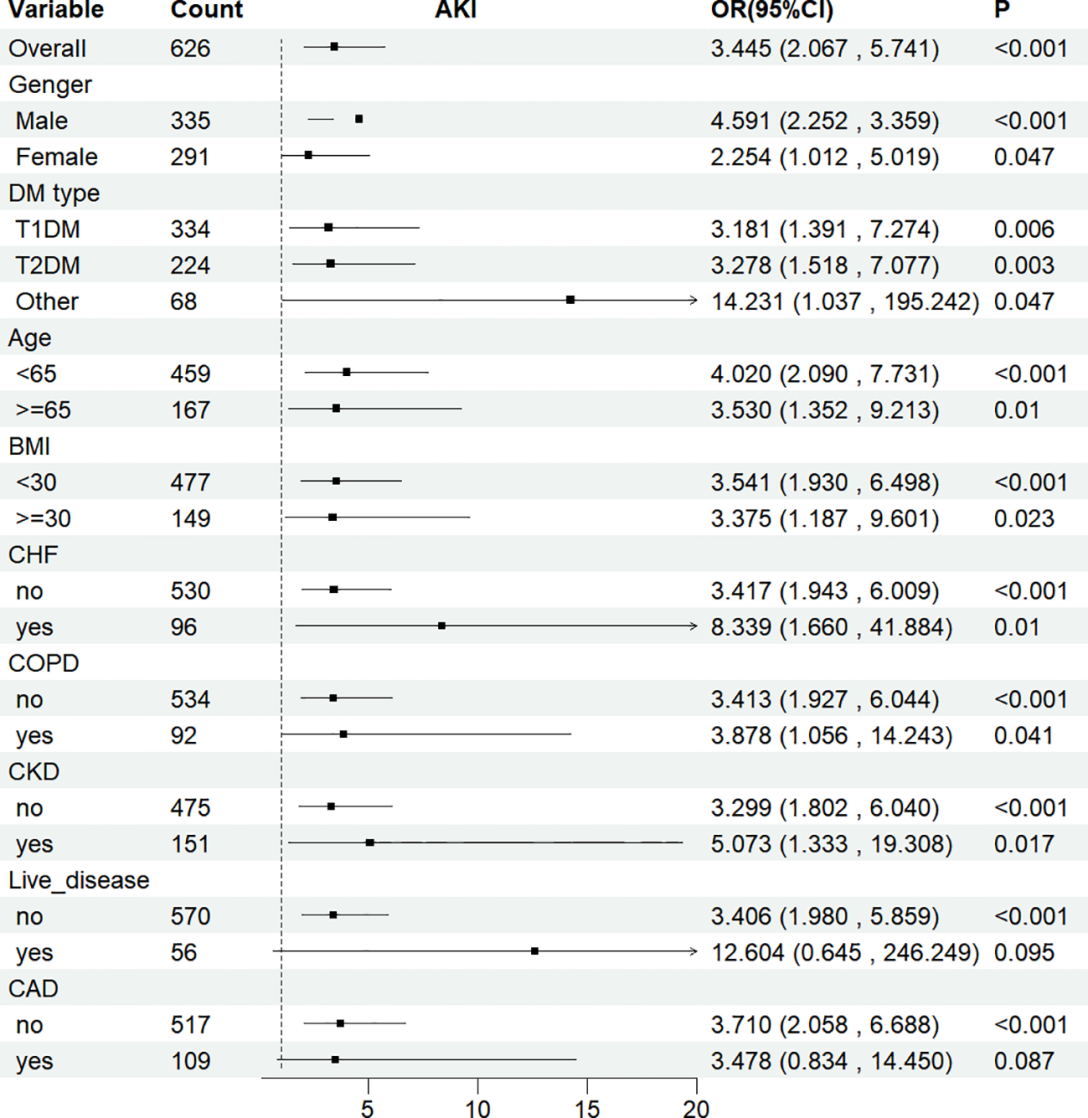
Subgroup analyses for the association of SOFA score with AKI. OR, odds ratio; CI, confidence interval. Subgroup analysis of SOFA and the risk of AKI in the fully adjusted model with SOFA as a Categorical variables (Q1 and Q2).

To further examine the relationship between SOFA scores and AKI incidence, RRT usage, and in-hospital mortality rates, stratified analyses were performed based on gender, age, BMI, DM type, CHF, COPD, CKD, liver disease, and CAD. Whether stratified by different genders (males = OR: 4.591, 95% CI: 2.252–3.359; females = OR: 2.254, 95% CI: 1.012–5.019), DM types (T1DM = OR: 3.181, 95% CI: 1.391–7.274; T2DM = OR: 3.278, 95% CI: 1.518–7.077; and other = OR: 14.231, 95% CI: 1.037–195.242) or BMI categories (<65 kg/m2 = OR: 3.541, 95% CI: 1.930–7.731; ≥65 kg/m2 = OR: 3.375, 95% CI: 1.187–9.601), the fully adjusted model showed a significant relationship between SOFA scores and AKI incidence. The same relationship was observed in patients with CHF, COPD, CKD, liver disease, and CAD (**Figure 6**). In the fully adjusted model with SOFA as a continuous variable, the direction of the relationship between the SOFA score and these outcomes—AKI incidence, RRT usage, or in-hospital mortality rates—remained consistent across different stratified analyses, indicating a similar correlation of SOFA scores across most subgroups (**Supplemetary Figure S3**).

## 4 Discussion

This study confirmed that the SOFA score is a crucial predictor of AKI in patients with DKA. Notably, this association remained statistically significant even after adjusting for all potential confounding factors. Our analysis demonstrated that higher SOFA scores that were also associated with RRT usage and increased in-hospital mortality. The two secondary outcome indicators—RRT usage and in-hospital mortality—further supported the reliability of the SOFA score in predicting the primary outcome, AKI. Subsequent analyses demonstrated a stable linear relationship, and these findings remained statistically significant in the fully adjusted model. We believe this study expands the application of the SOFA score to the diabetes domain, suggesting its potential as a clinical decision-making tool for treating patients with DKA. We found that each unit increase in the SOFA score was associated with a 35% increase in the risk of AKI incidence, a 43.5% increase in RRT usage, and a 43.7% increase in the risk of in-hospital mortality in patients with DKA. The SOFA score is crucial for assessing clinical death risk and prognosis in patients with sepsis (16) and is widely used to evaluate the clinical prognosis of critically ill patients in the ICU (17). Our findings align with previous studies, indicating that high SOFA scores are associated with increased mortality and complications in DKA patients. Thus, the SOFA score can effectively reflect the severity of illness in DKA patients, aiding in their clinical management. Furthermore, it is essential to closely monitor blood glucose levels, insulin resistance, and other indicators in patients with DKA with elevated SOFA scores to mitigate adverse outcomes. Despite adherence to current clinical guidelines, DKA remains a common and severe condition among patients with diabetes (18, 19), posing an escalating burden on global public health institutions. A 2006 prospective cohort study found that patients with cardiovascular failure, as defined by the SOFA score, had odds of death and adverse neurological outcomes 14.7 times (95% CI: 5.9–36.3) and 7.6 times (95% CI: 3.5–16.3) higher, respectively, compared to those without cardiovascular failure (20). Additionally, a 2021 meta-analysis indicated that a higher SOFA score is associated with an increased dialysis dependency risk (21). This underscores the importance of the SOFA score in clinically assessing AKI risk in patients in the ICU.

However, this study has limitations. First, as an observational study, we cannot establish a definitive causal relationship between SOFA scores and outcome indicators, despite employing rigorous statistical analyses. Second, we recorded only the initial SOFA scores at admission and did not track changes in SOFA scores throughout the follow-up period. Consequently, we may not have fully captured the dynamic changes in the status of various critical organs. Third, due to the inherent limitations of the MIMIC database, we could not ascertain the severity of certain conditions, such as heart failure, liver disease, and cancer, among other comorbidities. Additionally, relevant socioeconomic information for the patients was unavailable, which may have introduced potential biases in the outcomes. Fourth, further multicenter, prospective cohort studies are needed to validate our findings.

As machine learning techniques continue to advance, recent developments have introduced exciting new methodologies that can enhance predictive accuracy and model interpretability (22, 23). In this study, we employed a combination of Boruta, Random Forest, and SHAP models (24) for feature selection and data analysis, which demonstrated significant advantages in terms of interpretability and stability. Compared to the XGBoost and deep learning methods mentioned in recent literature, our approach excels in handling smaller datasets while ensuring model robustness. Specifically, the Boruta algorithm effectively identifies the most important features, reducing the dimensionality of the feature space and thus mitigating the risk of overfitting. The SHAP model further enhances interpretability by revealing the contribution of each feature to the final prediction, which is particularly crucial in medical data analysis.

While XGBoost and deep learning methods are well-suited for detecting complex patterns in large datasets, we believe our methods offer greater advantages in terms of flexibility, especially when dealing with smaller datasets commonly found in clinical settings. Deep learning models often require significant computational resources and large sample sizes, which may not always be feasible in resource-limited environments, such as hospitals. In contrast, Random Forest and Boruta can provide stable and accurate results even with smaller datasets, making them more practical and adaptable for real-world healthcare applications.

Furthermore, with the emergence of reinforcement learning (25), graph neural networks (GNNs) (26), and self-supervised learning (27), the potential for clinical data analysis has expanded significantly. Reinforcement learning can optimize treatment plans by dynamically adjusting medication dosages through interaction with the environment. Graph neural networks effectively capture complex patient relationships, especially when clinical data exhibit graph structures. Self-supervised learning provides innovative solutions for domains with unannotated data, reducing reliance on manual labeling. These emerging methods offer new directions for clinical decision support systems and personalized medicine, warranting further exploration and application.

Therefore, we believe that integrating machine learning methods, such as those used in our study, can significantly enhance clinical decision-making. As demonstrated in our analysis, these methods are effective in identifying key predictors of outcomes in DKA patients, enabling more targeted interventions and improving patient prognosis.

## 5 Conclusion

Our study applied the SOFA score to patients with diabetic ketoacidosis (DKA), emphasizing its utility as a vital clinical tool for this population. We demonstrated that the SOFA score not only reflects the severity of DKA but also reliably predicts the occurrence of acute kidney injury (AKI), the need for renal replacement therapy (RRT), and in-hospital mortality. This is particularly significant given the high incidence of AKI in DKA, a serious complication that exacerbates patient outcomes. Specifically, our findings showed that each unit increase in the SOFA score corresponds to a 35% greater risk of AKI, a 43.5% increase in RRT use, and a 43.7% rise in mortality, solidifying the SOFA score as an important early risk stratification and management tool for clinicians treating DKA patients.

Furthermore, our analysis confirmed that the SOFA score’s predictive power remains strong even after adjusting for multiple confounding factors such as demographics, laboratory parameters, and underlying comorbidities. This reinforces its applicability across various clinical settings, including subgroups with conditions like chronic kidney disease (CKD), heart failure, and other comorbidities that are often present in patients with DKA.

Beyond its established use in critically ill ICU patients, the extension of the SOFA score to the diabetes domain is an important contribution of our study. Our results suggest that the SOFA score could be highly beneficial in identifying high-risk DKA patients early, potentially guiding more targeted interventions to prevent AKI, optimize resource utilization, and lower mortality rates. Given the growing burden of DKA on public health, applying the SOFA score in this context could significantly improve clinical decision-making and patient outcomes.

## Data Availability

The original contributions presented in the study are included in the article/**Supplementary Material**. Further inquiries can be directed to the corresponding author.

## Supplementary material

Supplementary Figure 1 The cumulative event incidence curves for incidence of RRT.

Supplementary Table 1 Cox proportional hazards models of SOFA and AKI.

Supplementary Figure 2 Restricted cubic spline (RCS) showing the relationship between SOFA and AKI based on the fully adjusted Cox risk model.

Supplementary Figure 3 Subgroup analyses for the association of SOFA score with AKI, RRT and in-hospital mortality. Subgroup analysis of SOFA and the risk of AKI in the fully adjusted model with SOFA as a continuous variable.
