# Supplementary figures and images for: Association between SOFA score and risk of acute kidney injury in patients with diabetic ketoacidosis: an analysis of the MIMIC-IV database
Source: Front Endocrinol (Lausanne). 2024 Dec 23;15:1462330. doi: 10.3389/fendo.2024.1462330 (PMC11700799; doi:10.3389/fendo.2024.1462330)

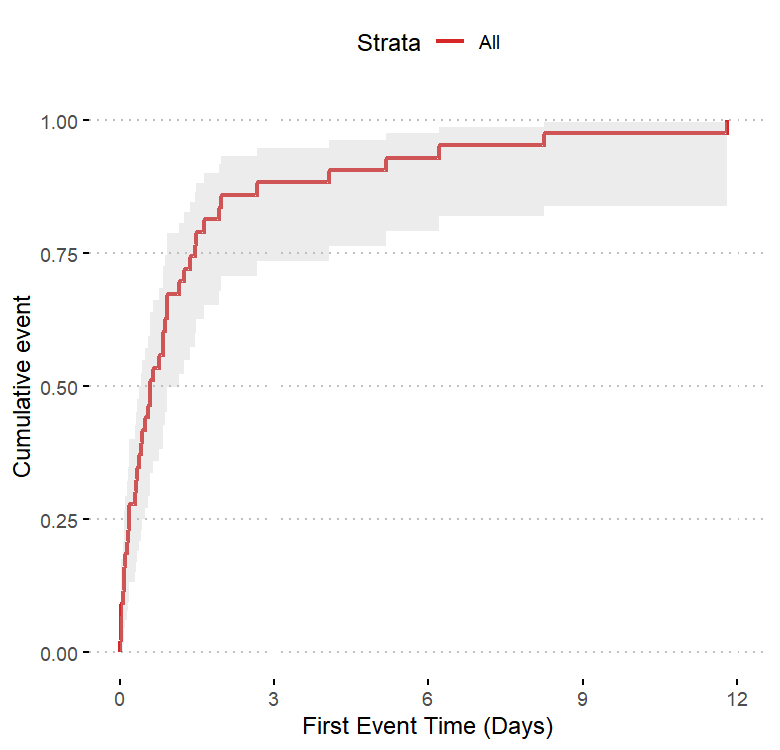

Supplement: Supplementary Figure 1 — The cumulative event incidence curves for incidence of RRT. [file Image1.tiff]

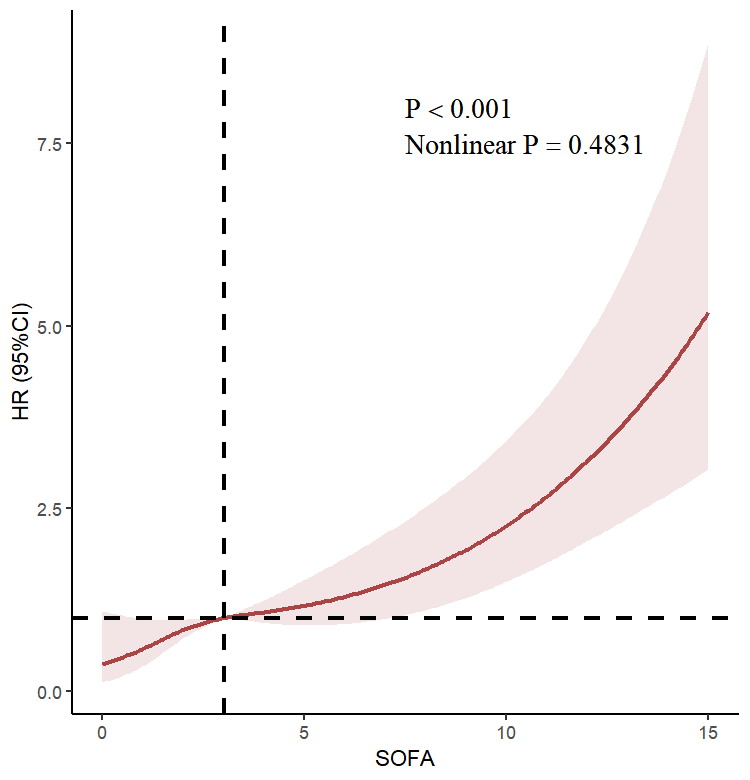

Supplement: Supplementary Table 1 — Cox proportional hazards models of SOFA and AKI. [file Image2.tiff]

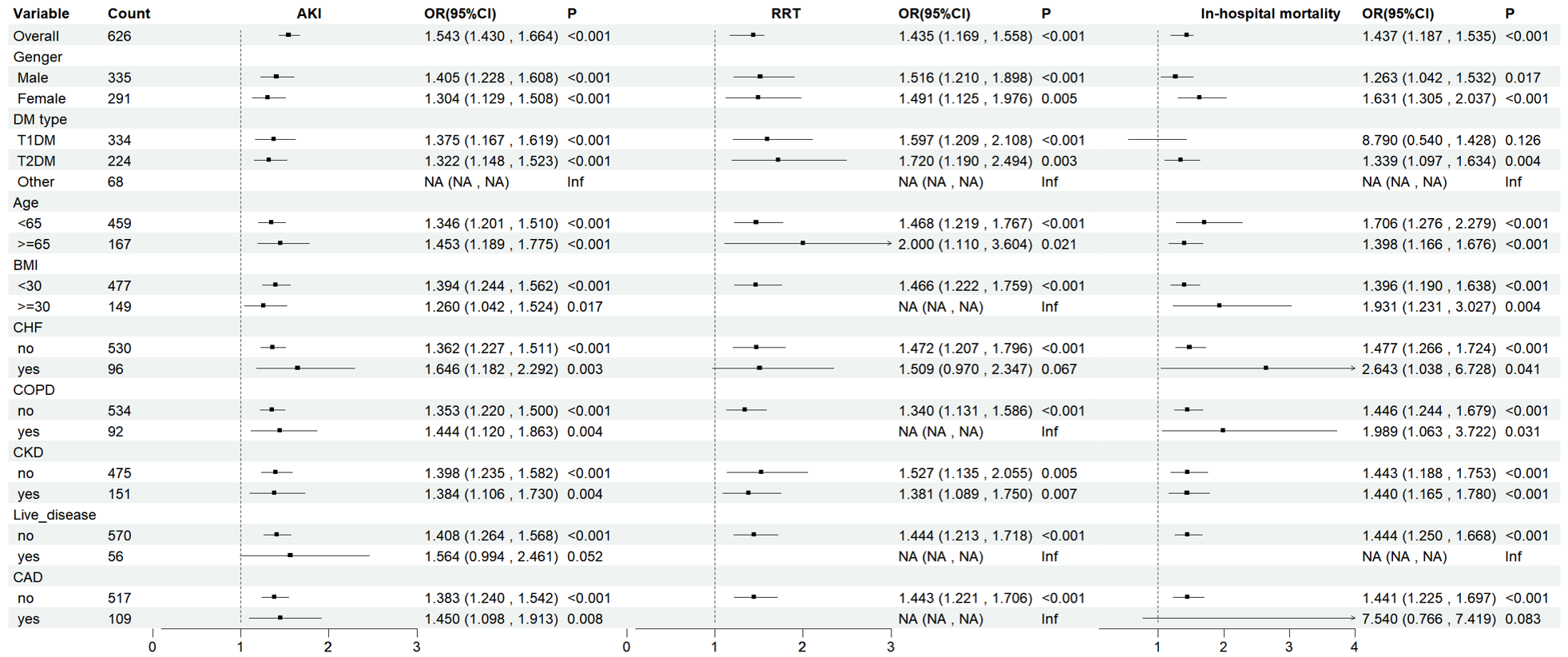

Supplement: Supplementary Figure 2 — Restricted cubic spline (RCS) showing the relationship between SOFA and AKI based on the fully adjusted Cox risk model. [file Image3.tiff]

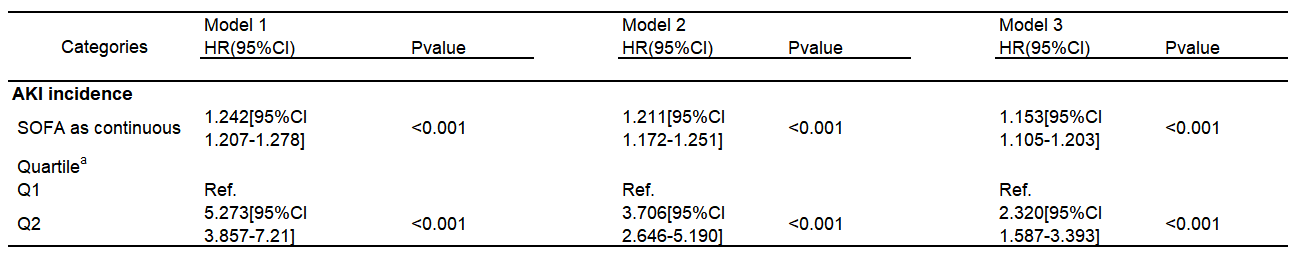

Supplement: Supplementary Figure 3 — Subgroup analyses for the association of SOFA score with AKI, RRT and in-hospital mortality. Subgroup analysis of SOFA and the risk of AKI in the fully adjusted model with SOFA as a continuous variable. [file Image4.tiff]
